# Report of Three Cases of Vesico-Vaginal Fistula

**Published:** 1871-08

**Authors:** A. Reeves Jackson

**Affiliations:** 785 Michigan Avenue; Chicago, Ill.


ARTICLE II.—*Report of Three Cases of Vesico-Vaginal Fistula.* By A. REEVES JACKSON, M.D., Chicago, Ill.

CASE I. *Vesico-Vaginal Fistula—Operation—Cure.*

Mrs. G., the wife of a farmer, was delivered, at the age of thirty-seven years, of her first child, after a labor of twenty-four hours, without instrumental aid. The child was still-born. Four years afterwards she again became pregnant, and, on the 20th of March, 1859, after being in labor thirty-six hours, was delivered, with the aid of forceps, of a second still-born child, at full term. About the fifth or sixth day she observed that she had no longer power to retain urine, except in very small quantities, and only in certain positions of the body.

An examination made some months subsequently revealed a fistulous opening of an ovoidal shape, three-quarters of an inch long, connecting the vagina with the bas-fond of the bladder.

As the patient had a great dread of a cutting operation, an attempt was made to close the fistula by repeated applications of nitrate of silver. This having proved entirely unavailing, and the condition of the patient being truly deplorable, she at length consented to undergo any operative proceeding that offered a fair prospect of success.

There were two points of difficulty in the case, either of which might militate against the success of an operation. One was a very contracted vagina, and the other an exceedingly sensitive condition of the urethra. This latter was so great, indeed, that the attempt to introduce a catheter produced the most excruciating pain. By using the instrument daily, however, the sensibility of the part became lessened; so that she was able to permit its presence during gradually lengthening periods, until finally it gave no uneasiness whatever, and for a few days immediately preceding the operation, she allowed it to remain four or five hours daily. The constriction of the vagina was removed in some degree by the use, two or three times a week, of an expanding speculum, by means of which the parts were put upon the stretch half an hour at a time. A series of sponge tents of suitable size would doubtless have been more efficient, but for some unaccountable reason she objected to their employment.

I also instructed the patient in the manner of introducing and removing the catheter herself, and this I regard as a very important part of the treatment, for in most cases it seems necessary to the success of the operation for vesico-vaginal fistula that the catheter remain constantly in the bladder, in order to carry away the urine so soon as it reaches that organ, and thus prevent any possible accumulation from endangering the newly-formed adhesions. And although the sigmoid catheter is a self-retaining instrument, yet it may and not unfrequently does slip from the bladder. Such an accident may occur during sleep, from an involuntary muscular effort; or the instrument may become entangled in the bedclothing, or in a variety of other ways it may be withdrawn, and if the patient be unable to replace it, and the medical attendant not near at hand, this circumstance alone may be sufficient to prevent success. Hence the importance of the patient being able to perform this operation without assistance, and likewise of realizing the necessity for having the catheter in the bladder during the entire progress of the after-treatment.

*Operation.*—The patient having the day before taken a full dose of castor oil, the operation was performed on the 12th of September, 1860, in the presence and with the assistance of Drs. Grattan, Bond and Bush, and Mr. Horace De Young, medical student. She was placed upon a table in the knee-elbow position, the pelvis being supported by a low stool covered with a pillow. By means of a Sims' speculum, the fistula was brought into tolerably good view, although, from the narrowness of the vagina, this was not easily done. The day, too, was unfortunately a cloudy one, a circumstance which added greatly to the difficulties of the operation, for I know of none in which a bright sunlight is more desirable than this.

However, the edges of the fistula were at length satisfactorily pared, a complete circlet being removed, having its edge strongly beveled at the expense of the vaginal mucous membrane and subjacent tissue. The instruments used in this stage of the operation were a long-handled tenaculum and a straight knife.

The patient was now released from her uncomfortable position, and allowed to rest until all bleeding from the cut surfaces should cease, and, in the meantime, I made preparations for the second stage of the operation.

Having the patient again placed in position, and the parts exposed, I introduced six metallic sutures, three of silver and three of iron wire, the points of insertion and emergence being each about half an inch from the edges of the opening. The ends of each pair being drawn tense, sufficient twist was given to them to approximate and maintain the edges of the fistula in close contact. A fenestrated leaden plate or button was then passed down to the wound, the six pairs of wires passing through the fenestrum. This latter was beveled on the side applied to the line of incision, which, since the tightening of the sutures, was perfectly straight. The plate was secured in its position by means of perforated cylinders of lead, one of which was passed respectively on each pair of wires, and having been pressed firmly against the plate while the wires were tightly drawn, they were firmly squeezed between the jaws of strong forceps. The wires were then cut off close to the cylinders, and the operation was finished.

A sigmoid catheter was introduced, and a full dose of McMunn's elixir of opium administered.

*After-treatment.*—Every morning and evening the patient took a full dose of opium, for the double purpose of preventing irritability and of constipating the bowels. This latter object was also aided by a diet consisting exclusively of crackers and tea. The catheter was removed daily for cleansing, and another inserted. At no time, however, did any troublesome incrustation form upon the instrument. The urine was received in a vessel placed under the catheter for the purpose. The vulva was carefully syringed three times a day with tepid water.

On the third day after the operation I examined the parts, and had the satisfaction of finding them in a most favorable condition. The button was firmly in its place, and no urine was perceptible in the vagina. On the tenth day I removed the button without difficulty, and found beneath it a firm, hard cicatrix, extending the entire length of the former fistula. The sutures were permitted to remain two days longer, at the end of which time they also were removed.

During the night of the twentieth day after the operation, and while the patient was sleeping, the catheter accidentally slipped from the urethra. She awoke with a strong inclination to pass water, and made the effort, when, to her inexpressible delight, she discovered that for the first time in eighteen months she had power to evacuate the bladder at will. She at once replaced the catheter, however, and continued to use it the next three or four days, allowing it to remain out of the bladder an hour the first day, two hours the second, and so on until she felt safe in dispensing with its use entirely.

CASE 2. *Vesico-Vaginal Fistula—Operation—After-treatment, complicated with vesical hæmorrhage—Cure.*

Mrs. Moore, the wife of an Irish laborer, aged thirty-four years, is the mother of four children. At the age of nineteen she gave birth to her first child, after a labor of eight days' (?) duration. No instruments were used. On the ninth day after her confinement she noticed the urine dribbling from her, and it has continued to do so down to the present time.

An examination, made June 1st, 1863, revealed a large fistula, irregularly elliptical in shape, with red thickened edges. It extended from about the middle of the vagina to the upper boundary of the anterior cul-de-sac. The cervix uteri pressed through the opening into the bladder. Her condition being one of uninterrupted misery, she gladly consented to an operation that promised any measure of relief.

*Operation.*—The patient having been suitably prepared, the operation was performed June 11, 1863. I had the assistance of Drs. Bond, Bush and Osmun. There were also present, Messrs. Boys, Williams and Gulick, medical students. Having placed her upon the elbows and knees, with the back towards a window, the vagina and fistula were exposed by means of a Sims' speculum. The cervix uteri was drawn away from the opening by means of a tenaculum, held by an assistant. The edges of the fistula were carefully pared, and brought together with eight silver wire sutures, each alternate one being clamped with perforated pieces of lead, and the others simply twisted. Previous to clamping the sutures, a fenestrated plate of lead was passed over the suture wires, as in the case of Mrs. G. A full dose of elixir of opium was given, the patient placed in bed, and a sigmoid catheter introduced.

*Progress of Cure.*—June 12th, 9 A.M. Patient did not sleep last night, but feels comfortable. The urine is scanty and bloody. It all passes into a vessel placed beneath the catheter. Syringe the vulva and vagina with cold water. Order crackers and tea exclusively as diet. Give a teaspoonful of elixir of opium.

9 P.M. The patient is very restless. Blood has been flowing all day, clogging the instrument, and necessitating its frequent removal. She complains of abdominal pain, and a constant desire to urinate. Remove the catheter, cleanse and replace it. Repeat opium.

13th, 9 A.M. Rested well last night, and is still drowsy. Pulse 104, soft and full. Syringe the vagina and vulva with cold water. Remove the catheter, and free it from clotted blood. The urine continues scanty and bloody. She is permitted to lie on her side.

7 P.M. Patient very uncomfortable, and complaining of an intense desire to urinate. No urine has flowed through the catheter for more than an hour. Remove the instrument, which is filled with a blood clot. A great deal of blood has been passing during the afternoon. Cleanse the instrument and replace it, giving exit to about two ounces of bloody urine, with great relief to the patient. The bleeding continues so profusely that it is found necessary to remove the catheter every few minutes. Accordingly I detail Messrs. Boys and Williams to remain with her during the night. At 10 P.M. I administer a full dose of opium, and enjoin the recumbent posture and strict quietude.

June 14th, 4 A.M. Am called in haste. The patient is in great pain from flatulent distension of the bowels. The catheter has been removed and cleansed every fifteen or twenty minutes during the night, on account of the continuous flow of blood. Pulse 100, skin cool, some thirst. Syringe the vagina with cold water, and order three grains of gallic acid to be given every two hours. Also give one-quarter grain of morph. sulph. Shortly after taking this latter she passed a large quantity of flatus from the bowels, and at once became easier.

10 A.M. Has taken two doses of the gallic acid. Bleeding from bladder much diminished. Has slept during the last four hours. Remove the catheter and cleanse it.

5 P.M. Feels very comfortable. Bleeding still less. Pulse 94. Cleanse and replace the catheter.

June 15th. Am called at 3½ A.M. The patient awoke with much pain, and withdrew the catheter herself. It was filled with clotted blood. She complains of great pain. The urinal contains a considerable quantity of bloody urine, and a heavy clot is in the bottom of the vessel. Introduce the catheter, and remove an ounce and a half of urine tinged with blood, giving great relief to the patient. Syringe the vagina with cold water, repeat the gallic acid, and enjoin strict rest.

10 A.M. She has taken the gallic acid every two hours. The bleeding continues. Once, on removing the catheter, a number of clots are forced from the urethra, sufficient to form a mass as large as a walnut.

1 P.M. She seems much better. The urine now flows through the catheter almost free from blood. Continue gallic acid.

8 P.M. Patient in good condition. Urine flowing nearly clear. Continue gallic acid, and give a quarter grain of morph. sulph.

12 Midnight. Patient quite comfortable. Leave her in charge of Mr. Boys.

June 16th, 8 A.M. She has passed a good night. Urine free from blood. Syringe the vagina. Change clothing and bedding.

8 P.M. Urine has continued to flow through the catheter, with only a tinge of blood. Repeat morphia.

11 P.M. Change catheter. Notice a very slight trace of blood in the urine.

June 17th, 8 A.M. Has slept well during the night. Urine has flowed freely, but still contains a small quantity of blood. Syringe vagina. Repeat morphia.

10 P.M. Has slept nearly all day. Change catheter.

June 18th, 8 A.M. Syringe vagina and change catheter. Repeat morphia.

From this time forward the patient felt comfortable, except from occasional pain caused by flatulence. A slight trace of blood continued in the urine, but was not sufficient in amount to clog the catheter.

June 22d. This morning, with the assistance of Messrs. Boys and Williams, I remove the plate and all the sutures except two. The parts appear well, and union perfect. Introduce catheter and remove a half ounce of urine, which has accumulated during the removal of the button.

June 23d. To-day she feels entirely well, and thinks she could walk home (a distance of eighteen miles).

June 24th. Examine the parts and remove the remaining sutures. Notice a small spot of ulceration at the site of one of the sutures. Touch it with nitrate of silver.

June 27th. Patient steadily improving. She sits up to-day an hour and a half without the catheter. Towards evening it is again removed two and a half hours.

June 28th. Bowels moved this morning spontaneously, the first time since the operation—a period of seventeen days. Remove the catheter four hours, and then allow the patient to urinate without assistance.

July 1st. She starts for her home perfectly well.

(She has since given birth to two children; one on Sept. 5th, 1864, and the second in 1867, both labors being natural, and not followed by any injury to the vagina.)

CASE 3. *Vesico-Vaginal Fistula, of 28 years' duration—Operation unsuccessful—A second operation also unsuccessful.*

Mrs. W., aged sixty-eight years, has been afflicted with vesico-vaginal fistula twenty-eight years. She has had eight children, and the accident occurred during her last confinement. The labor was a lingering one, and was completed at the end of three days by instruments. She attributes her misfortune to the improper use of forceps, and says that at the time of their application she felt something "pinching" her.

Her general health was good, and she submitted to operation November 10th, 1863. There were present Drs. Thomas Bond, of Hainesburg, N. J., and P. M. Bush, of Marshall's Falls, Pa., together with some of the female relatives of the patient.

The fistula, on being exposed, was found to be situated on the right side of the median line of the vagina, about eight lines in length, and four in breadth. It was deeply hidden in a sort of pouch caused by the surrounding hardened and elevated portions of the vaginal mucous membrane. The latter also presented some superficial ulcerations, one quite near the fistula. The uterus was prolapsed, and its cervix exhibited a small ulcer covered with an ash-colored slough.

*Operation.*—The edges of the fistula were pared by means of a straight knife, and then brought together and secured by seven silver wire sutures. The tubular needle with which I intended to introduce the sutures was unfortunately broken during the attempt to place the first stitch. Having no duplicate, I was obliged to use a curved needle, with a pair of ordinary tonsil forceps, which made the operation both tedious and difficult. I employed neither button nor shot. The wires were simply twisted, and cut off close to the line of incision.

At the conclusion of the operation, which lasted one hour and forty minutes, I gave a teaspoonful of McMunn's elixir of opium, had the patient placed in bed, and introduced a sigmoid catheter.

*Progress of treatment.*—10 P.M. She complains of some pain, and has a desire to urinate. Change the catheter and repeat the opium. The diet to consist exclusively of crackers and tea.

Nov. 11th, 9 A.M. Slept some during the night, and this morning feels comfortable. Change catheter, and syringe the vagina with cold water. Repeat opium.

9 P.M. Change catheter and repeat opium.

Nov. 12th, 9 A.M. Slept well all night. Change catheter, and find it a good deal incrusted with lime and clogged with mucus. Syringe vagina. The withdrawal and introduction of the catheter occasion much pain.

Nov. 18th. The vagina has been daily syringed with cold water, and the catheter changed twice daily. Since the operation I have observed that the cloths placed beneath the patient are always wet, but the nurse has accounted for this by saying that the urine overflows or flows back from the urinal. Notwithstanding this explanation, I have had misgivings that the operation has failed, and that the cloths referred to have been made wet by the urine which escapes through the fistula.

Nov. 19th. Am pretty confident this morning that I discover urine escaping from the vagina, while the catheter is temporarily removed for cleansing.

Nov. 20th. This morning remove the sutures, and discover that union has not taken place, and that the operation has failed. The patient expresses a determination to have it repeated.

Dec. 11. To-day I operate again, with the assistance of Drs. Bond and Bush. The mode of proceeding is the same as that before adopted, except that I use the fenestrated plate for steadying the sutures. At the conclusion of the operation, the patient gets a full dose of opium, which is repeated once or twice daily. The diet consists entirely of crackers and tea. The vagina is daily washed with tepid or cold water, and the recumbent position strictly observed. The catheter is kept in the bladder, being removed once daily for the purpose of cleansing.

On the fifth day after the operation I am satisfied that urine escapes from the fistula, and that the operation has again failed. Next day remove the plate and sutures. The size of the fistula has diminished nearly one-half, but the patient has no more power to retain the urine than before.

785 Michigan Avenue.

